# Molecular study on recombinant cold-adapted, detergent- and alkali stable esterase (EstRag) from *Lysinibacillus* sp.: a member of family VI

**DOI:** 10.1007/s11274-022-03402-5

**Published:** 2022-09-07

**Authors:** Amira A. Matrawy, Ahmed I. Khalil, Amira M. Embaby

**Affiliations:** 1 Environmental Studies Department, Institute of Graduate Studies and Research, Alexandria University, 163 Horreya Avenue, P.O. Box 832, Chatby, 21526 Alexandria Egypt; 2 Biotechnology Department, Institute of Graduate Studies and Research, Alexandria University, 163 Horreya Avenue, P.O. Box 832, Chatby, 21526 Alexandria Egypt

**Keywords:** *Lysinibacillus* sp., Recombinant esterase, Family VI, Cold-adapted, Detergent stable, Cu^2+^ resistant

## Abstract

Cold-adapted esterases have potential industrial applications. To fulfil the global continuous demand for these enzymes, a cold-adapted esterase member of family VI from *Lysinibacillus* sp. YS11 was cloned on pET-28b (+) vector and expressed in *E. coli* BL21(DE3) Rosetta cells for the first time. The open reading frame (654 bp: GenBank MT120818.1) encodes a polypeptide (designated EstRag: 217 amino acid residues). EstRag amino acid sequence has conserved esterase signature motifs: pentapeptide (GFSQG) and catalytic triad Ser^110^-Asp^163^-His^194^. EstRag 3D predicted model, built with LOMETS3 program, showed closest structural similarity to PDB 1AUO_A (esterase: *Pseudomonas fluorescens*); TM-align score program inferences. Purified EstRag to 9.28-fold, using Ni^2+^affinity agarose matrix, showed a single protein band (25 kDa) on SDS-PAGE, *Km* (0.031 mM) and *Kcat/Km* (657.7 s^−1^ mM^−1^) on *p*-NP-C2. Temperature and pH optima of EstRag were 35 °C and 8.0, respectively. EstRag was fully stable at 5–30 °C for 120 min and at pH(s) 8.0–10.0 after 24 h. EstRag activity (391.46 ± 0.009%) was impressively enhanced after 30 min preincubation with 5 mM Cu^2+^. EstRag retained full stability after 30 min pre-incubation with 0.1%(v/v) SDS, Triton X-100, and Tween-80. EstRag promising characteristics motivate performing guided evolution and industrial applications prospective studies.

**Supplementary Information:**

The online version contains supplementary material available at 10.1007/s11274-022-03402-5.

## Introduction

As one of the global major commercial and industrial enzymes, esterases (EC 3.1.1.1) and lipases (EC 3.1.1.3) are a family of hydrolases that are responsible for the hydrolysis and synthesis of acylglycerols (Anthonsen et al. 1995). They share several structural features and reaction processes, including as the α/β-hydrolase fold, the conserved catalytic triad (Ser-Asp/Glu-His), and the pentapeptide motif (G/A-X-S-X-G) (Arpigny and Jaeger 1999). Esterases favor short-chain substrates and follow conventional Michaelis–Menten law in terms of substrate specificity and kinetics (Chahiniana and Sarda 2009). Lipolytic enzymes have been widely used as industrial catalysts in chemical, pharmaceutical, cosmetics, food industry, laundry detergents, and environmental remediation due to their efficiency and some characteristics such as chemio-regio-selectivity, enantio-selectivity, cofactor independence and a wide substrate spectrum (Luisa Tutino et al. 2009; Kim et al. 2015; Romano et al. 2015).

Lipolytic enzymes maintain high level of activity even when subjected to extreme temperatures, pH, detergents or organic solvents, rendering them appropriate for use in harsh industrial practices (Luisa Tutino et al. 2009; Ma et al. 2013). Sustaining activity at low temperatures necessitates structural flexibility that allows substrates to be accommodated under these conditions (Marx et al. 2007). Cold-adapted/active esterases with high catalytic efficiency at low temperatures (spanning from 4 to 40 °C) (Sarmiento et al. 2015a; Jiang et al. 2016; Noby et al. 2018) are getting prominence due to their potential applications in food manufacturing, biocatalysts for the generation of temperature-labile products, pollution bioremediation in cold environments and rapid deactivation at mild temperatures (Cavicchioli et al. 2011) which in turn is very beneficial for energy savings (Joseph et al. 2007).

The review of literature has a plethora of reports addressing multitude of recombinant cold-active lipases/esterases from various species like Est11 from *Psychrobacter pacificensi* (Wu et al. 2015), EstHIJ from *Bacillus halodurans* (Noby et al. 2019), Est700 from *B. licheniformis* (Zhang et al. 2018), P7-4 esterase from *Salinisphaera* sp (Kim et al. 2011), EstPc from *Psychrobacter cryohalolentis* K5^T^ (Novototskaya-Vlasova et al. 2012), EstDR4 from *Deinococcus radiodurans* (Zhang et al. 2021) and EstO from *Pseudoalteromonas arctica* (Khudary et al. 2010). Nonetheless, in order to meet the ever-increasing needs of global enzyme markets, lipases/esterases with unique catalytic properties and robust stability under harsh conditions are urgently required and worthy searching for.

The microbial screening approach is a straightforward approach for discovering novel robust esterases in extremophiles microorganisms (e.g., psychrophilic, psychrotolerant, alkalophilic, halophilic, and so on) that live in extreme environments (van Rossum et al. 2013; Sarmiento et al. 2015b). However, the inability to meet the nutritional needs of 99% of the microbial flora in any habitat, as well as low enzyme output from culturable extremophiles, are two important roadblocks to novel enzyme discovery. Researchers across the world can now use a sequence-based screening strategy to screen a large number of microbial genomes on account of the rapid advancement of bioinformatics technologies. Currently, the GenBank database contains a large number of fully or partially annotated whole genome sequences of various microbial species. As a result, the sequence-based screening method from the GenBank database is regarded as a potent, time-saving, and cost-effective tool for discovering novel enzymes from extremophile microbial genomes.

*Lysinibacillus* is a psychrotolerant (Rizvi et al. 2021) newly re-classified genus of *Bacillus.* Due to changes at the genetic level and in the composition of the peptidoglycan in their cell walls, which included lysine, advances in bacterial taxonomy studies led to this modification in classification (Ahmed et al. 2007). The genus *Lysinibacillus* has attracted attention in recent years due to its biotechnological potential in the control of human life-threatening insects (Ahsan and Shimizu 2021), production of important biomolecules/enzymes with industrial prospects (Mechri et al. 2017), and environmental bioremediation (e.g., detoxifying a variety of pollutants like heavy metals and petroleum) (Jinal et al. 2019).

Remarkably, at the time of writing this article, a search of the literature databases found that both native and recombinant esterases from *Lysinibacillus* spp. have not yet been investigated. This has addressed the urgent need to mine the *Lysinibacillus* genome extensively in order to uncover esterases with potentially robust characteristics. In the current study, mining the whole genome sequence (GenBank: NZ_CP026007.1) of *Lysinibacillus* sp., YS11 as a psychrotolerant bacterium yielded 32 hits (open reading frames encoding nucleotide sequences) of putative esterases. As a result, the unstudied *Lysinibacillus* esterases and the psychrotolerance character of *Lysinibacillus* necessitated the selection of one esterase hit (locus WP_103118438.1) at random from the genome sequence NZ_CP026007.1 of *Lysinibacillus* sp. YS11 for cloning and heterologous expression.

In the context of the above-mentioned, the goal of this study is to clone, express, and characterize a novel cold-adapted esterase from *Lysinibacillus* sp. YS11. To the best of the authors' knowledge, this is the first piece of research to address the cloning, expression, and characterization of esterase from *Lysinibacillus* sp.

## Materials and methods

### EstRag construct, bacterial strain cultivating conditions, vectors, and chemicals

An open reading frame (ORF) (654 bp and 217 amino acids) from *Lysinibacillus* sp. encoding a novel cold-adapted esterase (EstRag) was artificially synthesized by GenScript Biotech ® CO., USA. pET-28b ( +) was used as the expression vector. Whilst *E. coli* BL21 (DE3) Rosetta (Promega Co., USA) was utilized as the cloning and expression host in this study. Lauria-Bertani (LB) broth was used for the activation and growing purposes of *E. coli* (BL21) DE3 Rosetta strain with an agitation speed of 180 rpm, at 37 °C for overnight. Substrates (Sigma-Aldrich Co., St Louis, USA) used for the enzyme assay were *p*-nitrophenyl acetate (*p*-NP-C2), *p*-nitrophenyl butyrate (*p*-NP-C4), *p*-nitrophenyl caproate (*p*-NP-C6), *p*-nitrophenyl caprylate (*p*-NP-C8), and *p*-nitrophenyl laurate (*p-*NP-C12). Imidazole was purchased from Loba Chemie PVT, Mumbai, India. Isopropyl-β- D-1-thiogalactopyranoside (IPTG), protein ladder, and kanamycin were purchased from Bioline, USA.

### Synthesis of recombinant plasmid pET-28a ( +)/EstRag

The ORF encoding the esterase gene from *Lysinibacillus* sp. YS11 was retrieved from GenBank. This ORF spanned from c2955726 to 2956379 nucleotides in the genome of *Lysinibacillus* sp. YS11 with the accession number NZ_CP026007.1.

The protein ID reference sequence for the esterase gene locus was WP_103118438.1. The retrieved nucleotide sequence of the esterase gene had a length of 650 bp. This nucleotide sequence encoding a novel cold adapted esterase (EstRag) was synthesized by GenScript Biotech®. Co., USA (U3326EL100 _4). The chemically synthesized esterase gene (654 bp) was cloned onto pET-28b ( +) through the restriction sites 5′Nco1/HindIII3′. The construct was nominated as pET-28b ( +)/EstRag.

### Transformation of pET-28b ( +)/EstRag into *E. coli* BL21 (DE3) Rosetta

The recombinant construct pET-28b ( +)/EstRag was transformed into chemically competent *E. coli* BL21 (DE3) Rosetta cells as stated by a previously reported protocol (Maniatis 1989).

### Recombinant EstRag expression in *E. coli* BL21 (DE3) Rosetta

The transformants *E. coli* BL21 (DE3) Rosetta cells carrying the construct pET-28b ( +)/EstRag were cultured in a 1L Erlenmeyer flask containing 200 mL of LB broth supplemented with kanamycin at a final concentration of 34 μg/mL Then, the culture was incubated at 37 °C with an agitation speed of 180 rpm until reaching an optical density of 0.6–0.8 at 600 nm. After that, 1 mM isopropyl -D-1-thiogalactopyranoside (IPTG) was added to the culture, and the culture was incubated for a further 18 h at room temperature (22 °C) and 180 rpm. After incubation, the induced cells were harvested by centrifugation at 6,000×g for 20 min at 4 °C and resuspended in 50 mM Tris–HCl buffer, pH 8.0. A previously described technique (Abady et al. 2021; Mahmoud et al. 2021) was applied to break down the induced cells. Concisely, the cell pellets were suspended in 4 mL of disruption buffer (50 mM Tris/HCl, pH 7.6; 50 mg/mL lysozyme, and 300 mM NaCl). Then, the mixture was incubated for 30 min at 37 °C with gentle shaking. Cell disruption was accomplished via sonication at 14,000 Hz (Fisher Brand TM Sound Enclosure, Thermo Fisher Scientific Co., USA) for five cycles of 25 s each, with a one-min pause on ice between the successive cycles. Cell debris was removed by centrifugation at 8400×g for 15 min at 4 °C. In new Eppendorf tubes, the soluble supernatant of the cell lysate was transferred and then preserved at − 20 °C until further analyses.

### Purification of recombinant expressed EstRag

Purification of the recombinant expressed EstRag was carried out using a procedure that has been previously described with minor modifications (Mahmoud et al. 2021). In brief, the resultant soluble portion of cell lysate containing 100 mg of crude protein was loaded onto a 2 mL Ni^2+^ -NTA affinity matrix. Unbound proteins were stripped away from the column by washing it with equilibration buffer (50 mM phosphate buffer, pH 7.5, containing 10 mM imidazole) with five times the bed volume until the absorbance at 280 nm reached zero. After that, washing the column with elution buffer (50 mM phosphate buffer, pH 7.5, containing 500 mM imidazole) eluted the bound 6-His-tagged recombinant EstRag protein. Eluted fractions with protein content (as verified by absorbance at 280 nm) were pooled and dialyzed by means of a dialysis bag with a 10 kDa MW cut off. at 4 °C for 24 h against 50 mM phosphate buffer, pH 7.5 with 3 times buffer exchange. Dialyzed recombinant EstRag activity was assessed using *p*-NP-C2 as a substrate.

### Protein content determination

The Bradford method (Bradford 1976) was used to determine the protein content of the crude soluble cell lysate and the purified fraction. Bovine serum albumin was used to develop a standard curve.

### SDS-PAGE

The crude cell lysate and all purified protein fractions resulting from the purification process were subjected to 10% sodium dodecyl sulfate polyacrylamide gel electrophoresis (SDS-PAGE) using the Laemmli method (Laemmli 1970). The molecular weight of recombinant EstRag was anticipated using a protein ladder.

### Recombinant EstRag esterase activity

As previously reported (Ma et al. 2015), enzyme activity was measured colorimetrically by estimating the quantity of released p-nitrophenol (*p-*NP) (from its absorbance at 410 nm) using *p*-NP-C2 as a substrate**.** A standard curve of *p-*NP was established to determine the extinction coefficient of *p-*NP. The reaction mixture (1 mL) contained p-nitrophenyl ester substrate at a final concentration of 0.5 mM, 50 mM Tris–HCl, pH 8.0, and recombinant purified EstRag unless otherwise mentioned. All enzyme assays were carried out in triplicate at room temperature unless otherwise stated. Under the indicated assay conditions, one unit of esterase activity is defined as the amount of enzyme that liberates one mol of *p*-nitrophenol per min.

### Kinetic parameters and substrate specificity determination

The specific activity of purified recombinant EstRag was assessed using five *p*-nitrophenol esters (*p*-nitrophenyl acetate (*p*-NP-C2,), *p-*nitrophenyl butyrate (*p*-NP-C4), *p*-nitrophenyl caproate (*p*-NP-C6), ρ-nitrophenyl caprylate (*p*-NP-C8), and *p*-nitrophenyl laurate (*p*-NP-C12)) as the substrates.

The initial reaction velocities with various concentrations (0.0015–3.0 mM) of *p*-NPAC2 were measured and then fitted to the Lineweaver–Burk transformation of the Michaelis–Menten equation to calculate Km and Vmax using Hyper32 Software. The kcat was also determined using the equation: kcat = Vmax/[E], where [E] is the total amount of EstRag in the reaction mixture.

### Biochemical characterization of recombinant purified EstRag

*p*-NPA was used as the substrate for all enzyme biochemical characterization assays. All reactions were carried out in triplicate. The values were provided as the mean of three replicates with standard error.

### EstRag pH and temperature optima

The optimal pH was established across a wide pH range of 5.0–11.0: pH 5.0–6.0: 50 mM citrate buffer, pH 7.0: 50 mM phosphate buffer, pH 7.6–9.0: 50 mM Tris–HCl buffer, pH 10.0–11.0: 50 mM glycine–NaOH buffer. All enzyme assays using different buffers were conducted at room temperature. The optimal temperature was established at several temperatures ranging from 5 to 60 °C. The control reaction was the enzyme activity evaluated without any pretreatment.

### Effect of temperature and pH on EstRag stability

The thermal stability of EstRag was investigated by measuring residual activity after incubating the enzyme at different temperatures (4.0–50 °C) in 50 mM Tris–HCl buffer, pH 8.0, at three-time intervals; 30, 60, and 90 min. After that, the reaction tubes were placed on ice for 5 min before performing enzymatic assays at an optimal temperature.

The influence of pH on EstRag stability was determined by incubating the enzyme at 4 °C overnight in the aforementioned buffers ranging from 5.0 to 10.0. Following the completion of the incubation period, enzyme assays were performed. At each pH, control reactions were carried out and residual activity was measured.

### Effect of metal ions, detergents, organic solvents, sodium chloride and inhibitors on EstRag stability

The influence of different metal ions on EstRag stability was estimated by incubating the enzyme in the presence of different metal ions; Ca^2+^, Mg^2+^, Fe^3+^, Mn^2+^, Cu^2+^, Zn^2+^, Mo^2+^, and K^+^ using two concentrations of 5.0 and 10 mM for each metal ion. The stability of recombinant EstRag in the presence of detergents was evaluated by incubating the enzyme with Tween 20, Tween 80, Triton X-100, and SDS at two concentrations, 0.1 and 0.25% (v/v %) for 30 min at 25 °C in 50 mM Tris-HCl buffer, pH 8.0. The influence of polar and non-polar solvents on recombinant EstRag stability was estimated using 10 and 20% (v/v) solutions of dimethyl sulfoxide (DMSO), acetone, butanol, isopropanol, glycerol, methanol, ethanol, and hexane. The effect of NaCl on EstRag stability was assessed by using salt concentrations ranging from 0.5 to 4.0 M after preincubation of EstRag with each NaCl concentration for 30 min at room temperature. The impact of β-mercaptoethanol and ethylene diamine tetra acetic acid (EDTA) on EstRag stability was investigated at concentrations of 5 and 10 mM for each.

In all investigations, the purified EstRag was pre-incubated for 30 min at room temperature in 50 mM Tris-HCl, pH 8.0, containing the above-mentioned agents at the relevant concentration. All enzymatic assays were carried out following the end of the incubation time. An enzyme test without pre-treatment was used as a control reaction. Values are presented as the mean of three replicates with SE.

### In silico EstRag sequence analyses

The N-terminal signal peptide of the EstRag amino acid sequence was predicted using the Signal IP 6.0 server (https://services.healthtech.dtu.dk/service.php?SignalP-6.0). The Expasy, Swiss Bioinformatics 13 Resource Portal (https://web.expasy.org/translate/) was used to obtain the translated protein amino acid sequence of EstRag. Using the BLASTN and BLASTP online programs, the nucleotide sequence of the *EstRag* gene and its translated protein amino acid sequence were searched against the non–redundant nucleotide collection database and UniProtKB/Swiss-Prot (Swissprot), respectively. The SAS server (https://www.ebi.ac.uk/thornton-srv/databases/sas/) was used to predict the secondary structure of the translated AXE-HAS10 protein. CLC Sequence Viewer 8.0 was used to align the EstRag amino acid sequence with that of other esterases from other species. The MEGA 11.0 software was used to build a phylogenetic tree portraying the evolutionary relationships of the aligned sequences. The selection of representative examples of esterases that would cover all available reported esterases (I-XIX) was considered according to the two classifications of esterases: Arpigny and Jaeger in 1999 (updated by Jaeger and Eggert in 2002, Hausmann and Jaeger (2010), and Kovacic et al. in 2019) and ESTHER database The online Local Meta-Threading Server (LOMETS3) located at the server (https://zhanggroup.org/LOMETS/) was used to predict the three-dimensional (3D) structure of the EstRag protein. TM-align (Quick & Accurate Structural Alignment) online program located at the server (TM-align: A protein structure alignment algorithm using a TM-score rotation matrix (zhanggroup.org) was used by LOMETS to match the first predicted 3D model to all structures in the PDB library. The predicted 3D structure model of EstRag was visualized by PyMOL (Schrödinger, LLC, Portland, OR). The online program ExPASy was used to estimate the theoretical isoelectric point (pI) and predicted MW of EstRag. Three programs were directed to predict the presence of transmembrane helices in the EstRag protein: TMHMM2.0 (https://services.healthtech.dtu.dk/service.php?TMHMM-2.0/). SOSUI (https://harrier.nagahama-i-bio.ac.jp/sosui/mobile/) and PHOBIUS (https://www.ebi.ac.uk/Tools/pfa/phobius).

## Results

### Cloning, expression, sequence analysis and phylogeny of EstRag

The full-length of esterase ORF (654 bp) from *Lysinibacillus* sp. YS11 reference sequence NZ_CP026007.1 of *Lysinibacillus* sp. YS11, with the protein ID: WP_103118438.1, was retrieved from GenBank and chemically synthesized by GenScript Co. The chemically synthesized esterase gene was cloned in pET-28b ( +) expression vector. The cloned esterase gene was successfully overexpressed in the *E. coli* BL21(DE3) Rosetta strain. The recombinantly expressed protein, which deduced 217 amino acid residues of the cloned esterase gene, was designated EstRag. The nucleotide sequence of the EstRag encoding gene was deposited in GenBank under the accession number MT120818.1. However, the translated amino acid sequence EstRag was given the protein ID: QIT07223.1. A BLASTp sequence similarity search against the non-redundant database revealed that the translated EstRag amino acid sequence had high similarity identities with esterases from *Lysinibacillus* sp.: 97.7% identity with WP_036076201.1 of *L. boronitolerans*, 95.85% identity with WP_205444398.1 of *Lysinibacillus fusiformis*, 93.09% identity with WP_054609366.1 of *Lysinibacillus* sp.ZYM-1, and 92.17% identity with WP_036127121.1 from *Lysinibacillus* sp. However, a BLASTp sequence similarity search against the Uniprot/Swiss-protein database and Protein Data Bank (PDB) revealed that EstRag had low similarity with the following carboxylesterases/thioesterases: 29.68% identity with Q53547.1 of *Pseudomonas fluorescens*, 29.55% identity with Q51758.1 of *P*. *fluorescens*, 26.94% identity with Q6CJK6.1 of *Kluveromyces lactis* NRRL-Y-1140, 23.61% identity with Q54T49.1 of *Dictyostelium discoideum*, 29.68% identity with 1AUO_A of *P*. *fluorescens*, 29.66% identity with 6BJE_A of Homosapiens, 28.98% identity with 2H1i_A of *Bacillus cereus*, 25.81% identity with 4H0C_A of *Daydobacter fermentum*, 25.23% identity with 3CN7 of *P.aeruginosa*, 24.02% identity with 4F21_A of *Francisella tularensis*, 23.56% identity with 4HFZ_A of *Cereibacter sphaeroides*, and 19.61% identity with 3DOH_A of *Thermotoga maritima*.

**Fig. 1:**
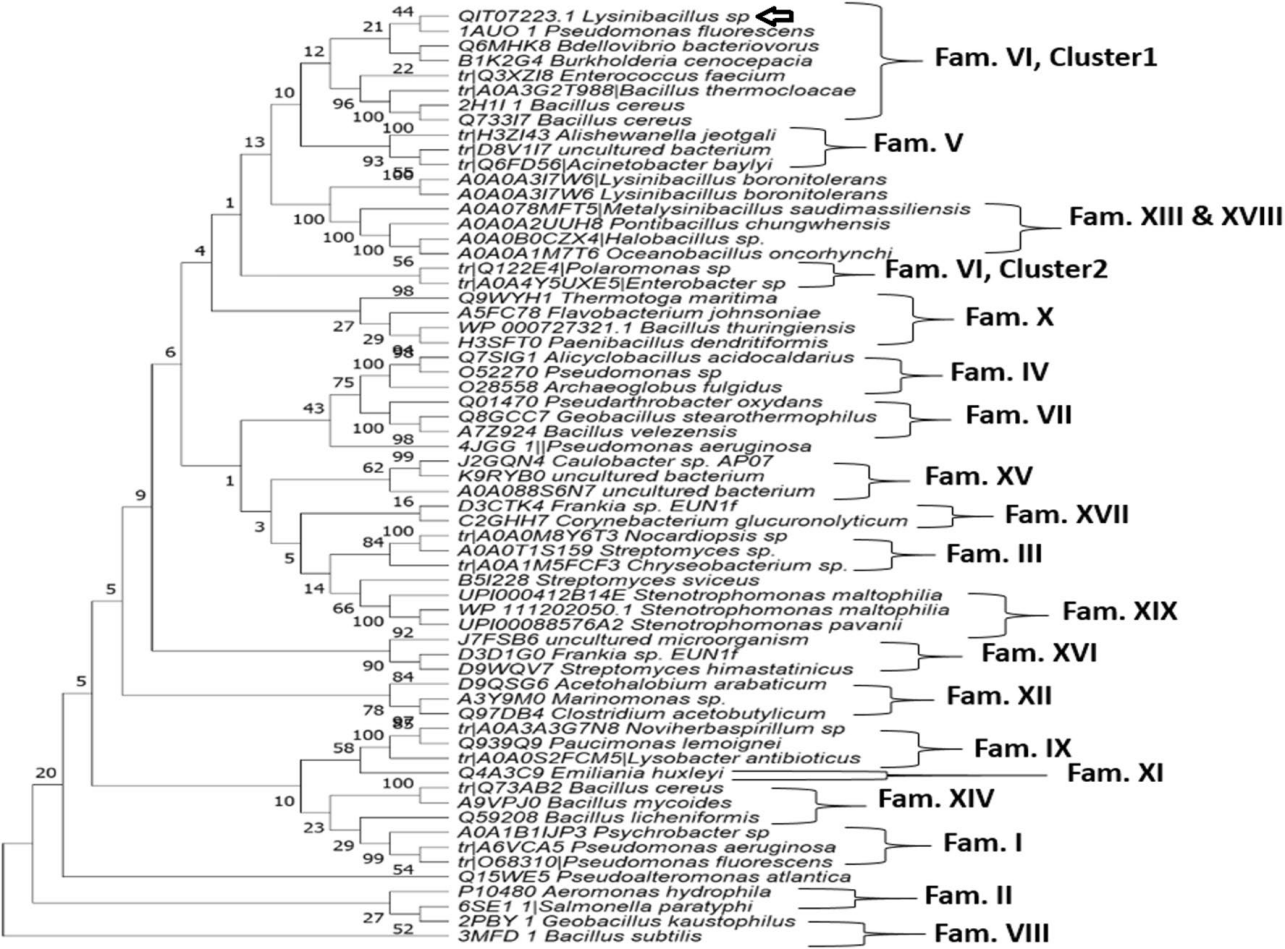
A Neighbor-Joining phylogenetic tree constructed by MEGA 11.0 shows the EstRag phylogenetic relationships in relation to esterases and lipases of 19 families (I-XIX) from other species. The rooted phylogenetic tree was constructed according to an alignment of full-length amino acid sequences of representative examples of esterases and lipases belonging to the currently available 19 families using the neighbor-joining method, the Jakes-Cantor model, built in MEGA 11.0. The empty arrow indicates EstRag. The bootstrapping value was set to be 1000. The bar indicates the branch length was 1.0. The accession numbers of esterases and lipases amino acid sequences, displayed on the tips of branches, were retrieved from the PDB, UniProtKB, and GenBank databases

A neighbor-joining phylogenetic tree (Fig. 1) including 63 esterases and lipolytic enzymes covering the previously classified 19 families (I-XIX), was constructed by MEGA software 11.0 in order to classify EstRag in relation to those families. The analysis of the constructed phylogenetic tree greatly suggested that EstRag was closely related to family VI esterases according to the classification of Arpigny and Jaeger 1999 (updated by Jaeger and Eggert 2002, Hausmann and Jaeger (2010), and Kovacic et al. 2019). Meanwhile, Family VI of esterases in ESTHER database (Esther Home Page (inrae.fr), the corresponding classification scheme of esterases, is nominated LYsophospholipase_Carboxylesterase.

**Fig. 2:**
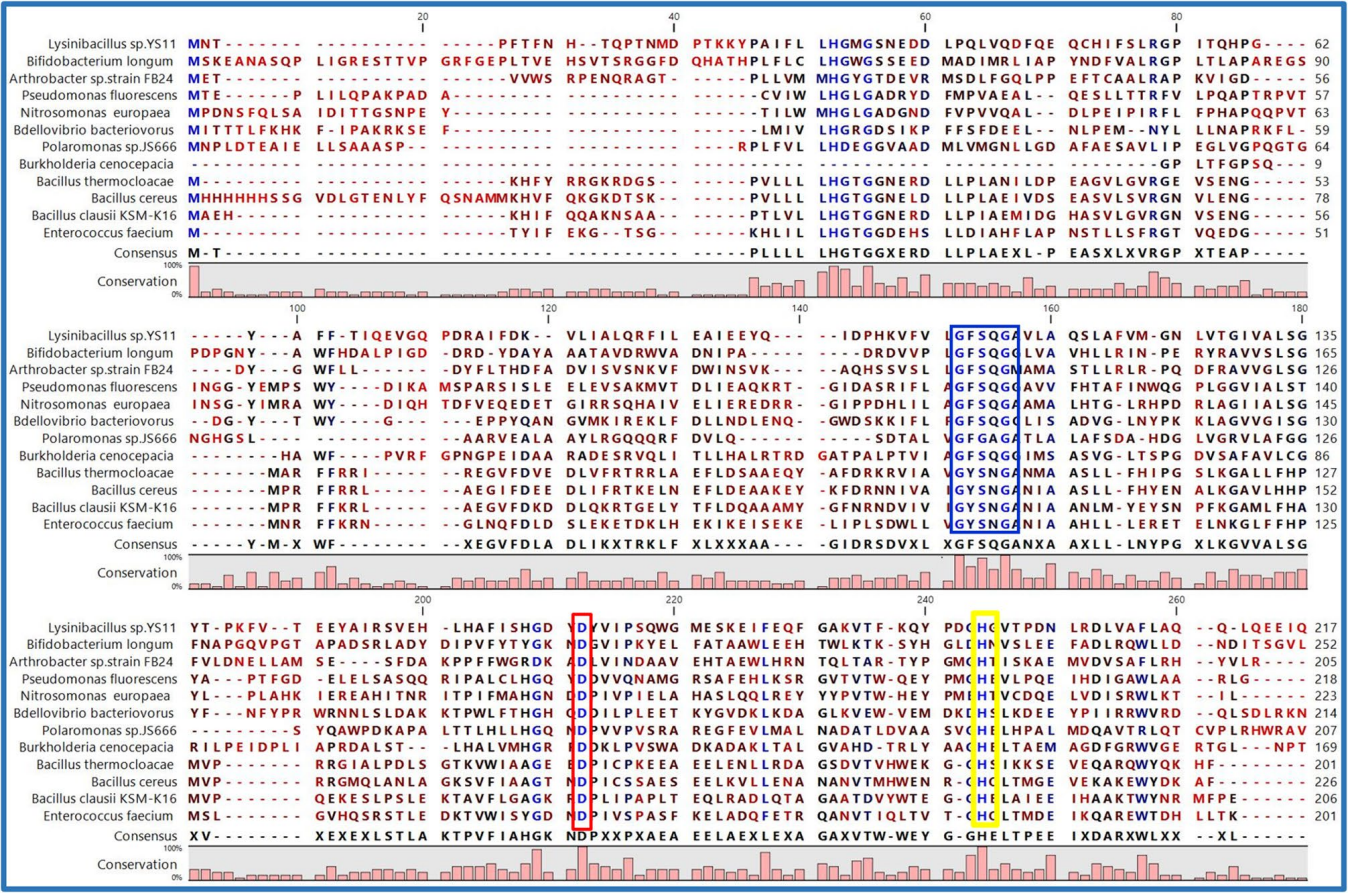
Multiple sequence alignment, performed by CLC Sequence Viewer 8.0, among some representative selected esterases and lipases of bacterial origin, belonging to Family VI, retrieved from the ESTHER database, along with EstRag of *Lysinibacillus* sp. YS11, shows the conserved signature features among these proteins. Penta motif (GXSXG): indicated by the blue rectangle including the catalytic Ser residue. Asp and His residues in the catalytic triad are indicated by red and yellow rectangles, respectively. The catalytic triad of EstRag was indicated at Ser^110^, Asp^163^, and His^194^. The accession numbers of the selected esterases and lipases were (QIT07223.1: EstRag of *Lysinibacillus* sp. YS11), (Q8G476: *Bifidobacterium longum*), (A0JRN6: *Arthrobacter* sp. strain FB24), (1AUO_1: *Pseudomonas fluorescens*), (Q820N9: *Nitrosomonas europaea* ATCC19718), (Q6MHK8: *Bdellovibrio bacteriovorus* strain ATCC15356),, (Q122E4: *Polaromonas* sp.JS666), (B1K2G4: *Burkholderia cenocepacia* strain MC0-3), (A0A3G2T988: *Bacillus thermocloacae*), (2H1I_1: *Bacillus cereus*), (Q5WGE5: *Bacillus clausii* KSM-K16), and (Q3XZI8: *Enterococcus faecium* strain ATCC BAA-472). Identical, similar, and unrelated amino acids residues along whole sequences were colored in blue, black, and red, respectively

Multiple sequence alignment revealed that the typical catalytic triad of active site serine (Ser110) in the pentamotif G-X-S-X-G, conserved aspartic acid (Asp163), and histidine (His194) residue motif was localized in EstRag and some representative members of bacterial esterasses and lipases from family VI (Fig. 2). EstRag's consensus pentamotif (GFSQG) covered residues 108–112.

SignalP6.0 analysis evidenced that EstRag's amino acid sequence lacks both the cleavage site and the N-terminal signal peptide. EstRag protein was not a transmembrane protein, according to TMHMM2.0, SOSUI, and PHOBIUS analyses.

### Structural modeling of EstRag

**Fig. 3:**
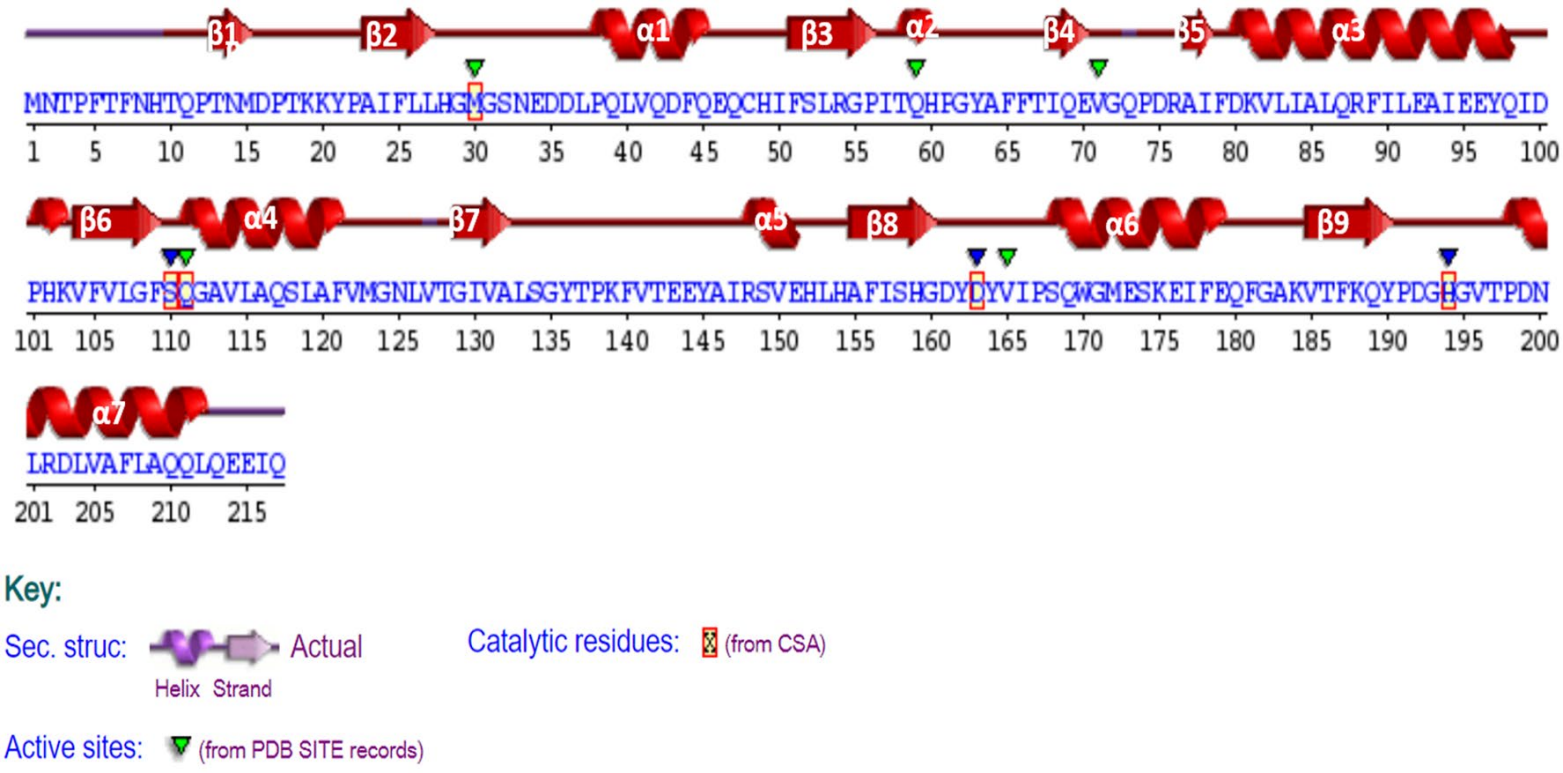
A Predicted secondary structure of EstRag as determined by SAS sequence annotated structure server using the PDB template 1AUO of *P*. *fluorescens* showing 9 β-sheets and 7α-helices along with the catalytic residues at Met^30^, Ser^110^, Gln^111^, Asp^163^, and His^194^ highlighted with red rectangles

The secondary structure of EstRag (Fig. 3) was predicted by the SAS online program using the esterase of *Pseudomonas fluorescens* (PDB entry 1AUO_A). The topological features of EstRag depicted in Fig. 3 reveal that EstRag is a typical α/β-fold hydrolase with 9 β-sheets and 7 α-helical structures. The Ser^110^ residue is found after β6, Asp^163^ is after β8, and His^194^ is after β9.

**Fig. 4:**
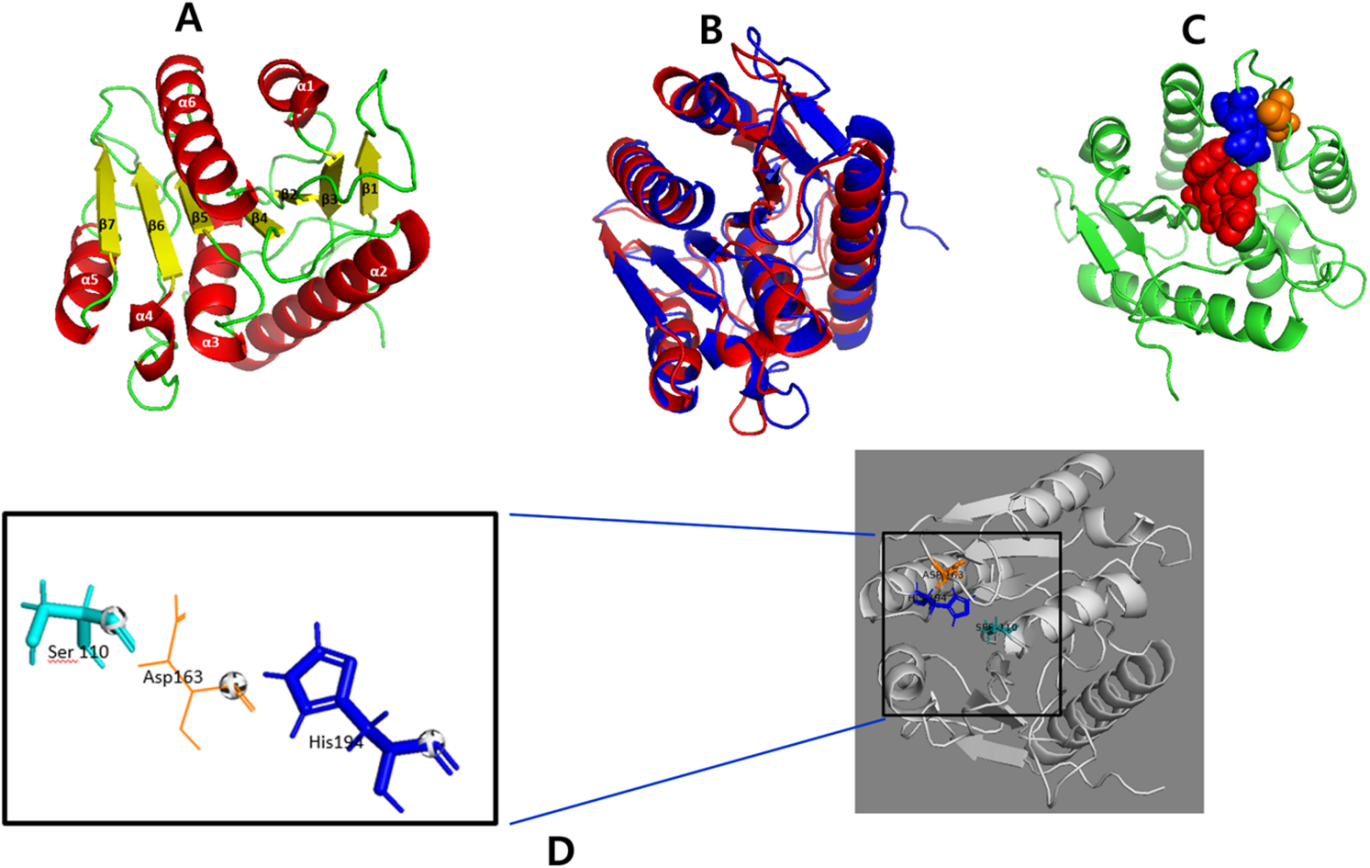
Initial 3D structure model of EstRag predicted by LOMETS local meta-threading server, version 3 server in cartoon views showing. **A** 7 β-sheets and 6 α-helices, **B** Superposition of EstRag 3D model (blue color) with PDB: 1AUO_1 (red color) of *P*. *fluorescens* as the protein structure template with an RMSD value of 2.13, **C** Penta motif signature feature of esterases (G-X-S^110^-X-G), Asp^163^, and His^194^ in red, orange, and blue spheres, respectively, and **D** Localization of the catalytic triad amino acid residues on loops joining α helices and β-sheets. PyMOL2 software was used to visualize the 3D structural predicted model

The three-dimensional structure of EstRag was modeled by the LOMETS3 online program using 10 top templates, selected from 110 templates, identified by 11 threading alignment methods. Among the top ten templates, the template PDB entry 1fj2_A (human acyl protein thioesterase) gave the best sequence alignment with EstRag amino acid sequence, with a normalized Z-score of 4.39. As a rule of thumb, a Z-score of threading alignment ≥ 1 indicates good alignment. The predicted 3D structure model of EstRag was shown in Fig. 4A. The 3D model structure of EstRag exhibited a typical α/β hydrolase fold with 7 β-sheets and 6 α-helices. The TM-align structure alignment online program revealed that the 3D structural model of EstRag exhibited the closest structural similarity to the top ten PDB templates. The PDB entry 1AUO_A: esterase of *P. fluorescens* had the closest structural similarity to EstRag and the highest TM-score of the top ten PDB structural analogue templates (0.853). When compared to the remaining 9 top PDB structural analogues templates, the superimposed 3D model of EstRag with PDB entry 1AUO_A (Fig. 4B) exhibited the lowest root mean square deviation (RMSD) value (2.13). The amino acid residues representing the catalytic triad of Ser^110^, Asp^163^, and His^194^ (Fig. 4C) were indeed localized in close proximity. The catalytic serine residue (Ser^110^) was positioned on a nucleophilic elbow joining β3- strand and α4- helix within the core structure, whilst Asp^163^ and His^194^ were positioned on loops between β5-α6 and β6-α7, respectively as shown in Fig. 4D.

As deduced from the bioinformatic analysis at the ExPASy online server, EstRag has a predicted molecular mass of 24.5 kDa and a theoretical pI of 4.95, respectively. Besides, EstRag has 26 negatively charged residues (Asp + Glu) and 13 positively charged residues (Arg + Lys).

### EstRag 3D model: structure validation and refinement

**Fig. 5:**
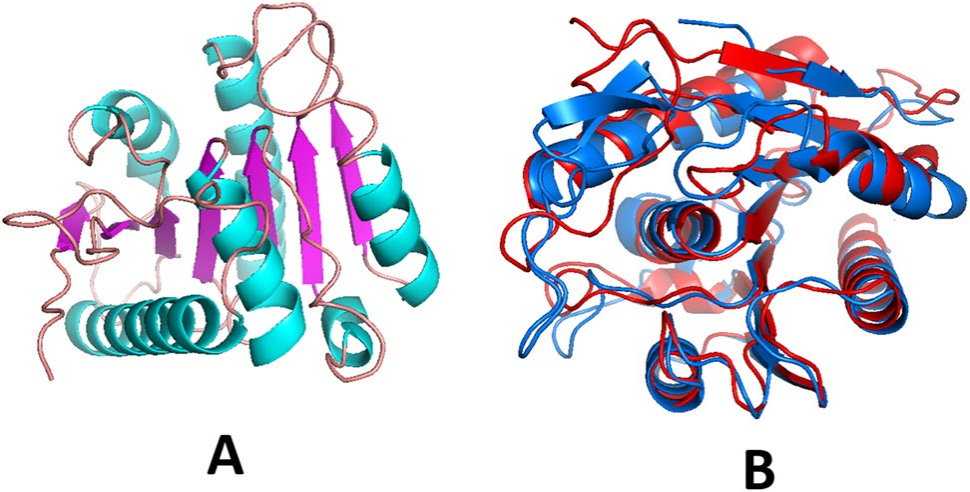
3D refined structure model of EstRag predicted by 3D.^refine^ Protein Structure Refinement Server in cartoon views showing. **A** Refined model of initial 3D structure model of Estrag and **B** superposition, performed by TM-align protein structure alignment of refined 3D structure model of EstRag (red color chain) with PDB: 1AUO_1 (marine blue color chain) of *P*. *fluorescens* as protein structure template with an RMSD value of 2.14

In order to validate the 3D structure model of EstRag, the initial model built by LOMETS and the refined 3D structure model built by 3D ^refine^ online program were evaluated by four estimates through the following online programs: SAVES 6.0 package (including three analyses PROCHECK, Verify 3D, and ERRAT) and PROSA. The results of these analyses were presented in supplementary fileS1 (Fig. 5)

### Expression and purification of recombinant EstRag

**Fig. 6:**
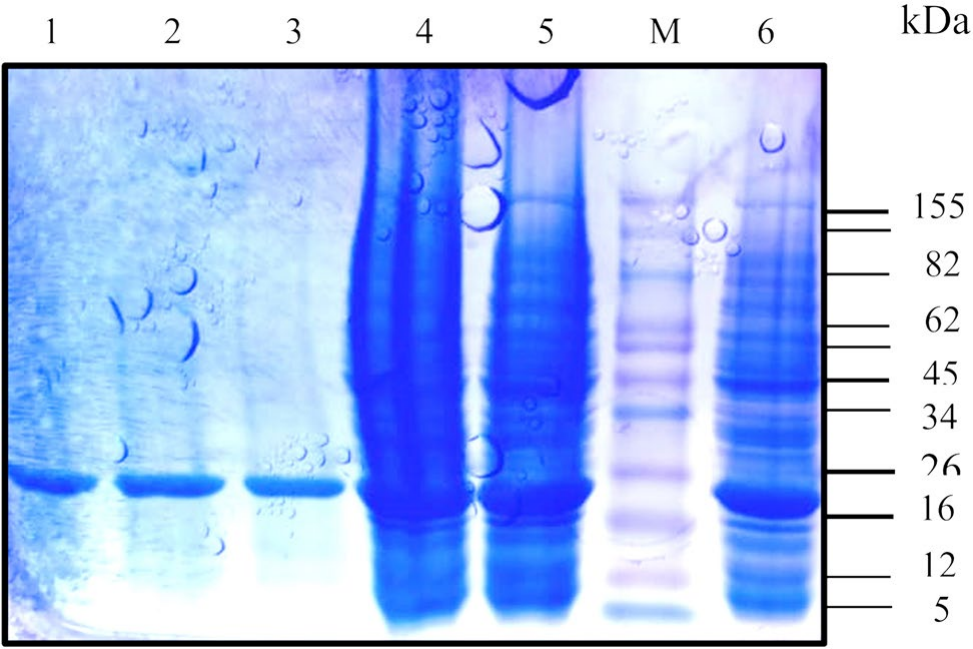
10%SDS-PAGE showing crude and purified to homogeneity EstRag. M: protein ladder. Lanes (1–3): purified to homogeneity EstRag after Ni^2+^—affinity chromatography step. Lanes (4–6): crude cell lysate of 1 mM IPTG induced recombinant *E. coli* BL21(DE3) Rosetta cells harboring the construct pET-28(b) + /EstRag

EstRag was overexpressed in 1 mM IPTG induced recombinant *E. coli* BL21(DE3) Rosetta cells harboring the construct pET-28b ( +) /EstRag at room temperature after 18 h of induction at 180 rpm. The recombinant EstRag, expressed as a fusion tag with 6-His residues on its C-terminus, was purified to homogeneity with Ni^2+^-agarose affinity chromatography (Table 1) with specific activity, fold purification, and yield of 23.08, 6.08, and 39.37, respectively. The purified to homogeneity recombinant EstRag displayed a single protein band with a molecular weight of around 25.0 kDa (Fig. 6).

**Table 1:** Purification table of recombinant EstRag using Ni^2+^-affinity agarose matrix

Purification step	Total units	Total mg protein	Specific activity (U/mg)	Fold	Yield (%)
Crude cell lysate	9500	40.8	232.84	1.00	100
After Ni^2+^-affinity agarose chromatography	3500	1.62	2160	9.28	36.84

### Biochemical characterization of recombinant EstRag

**Fig. 7:**
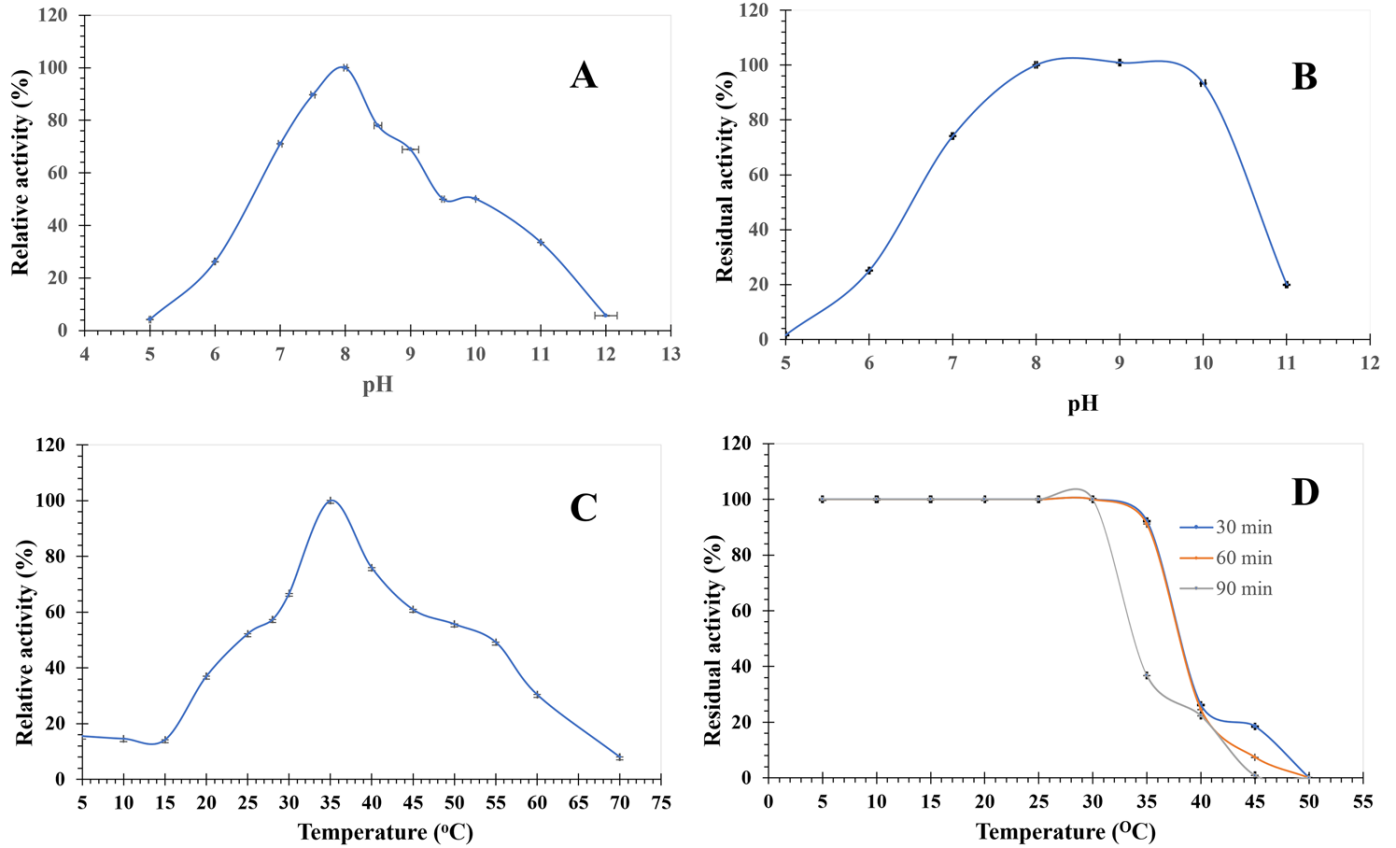
pH-temperature profile of purified to homogeneity EstRag. **A** EstRag activity profile over pH range 5–12. **B** pH stability profile of EstRag over pH range 5–11. **C** EstRag activity profile over temperature range 5–70 °C. **D** Temperature stability profile of EstRag over temperature range 5–50 °C. Results are the mean of experimental readings performed in triplicate ± SE (standard error) bars

The purified EstRag displayed cognizable activity over a wide range of pH (s) from 5.0 to 12.0. Significant differences (*P* < 0.05) were evidenced among values of enzyme activity over the tested range of pH (s). The optimum pH for enzyme activity was realized at pH 8.0 (Fig. 7A). Pertaining to pH stability, the purified EstRag exhibited 100, 100, and 93.41% stability for 24 h at pH (s) 8.0, 9.0, and 10.0, respectively (Fig. 7B). EstRag stability decreased significantly (*P* < 0.05) at pH(s) less than 8.0 and greater than 10.0. Regarding the enzyme-temperature profile, an appreciable enzyme activity with significant differences at *P* < 0.05 was remarked over a wide range of temperatures (5–60 °C). Whereas the optimal activity was achieved at 35 °C (Fig. 7C). EstRag full activity (100%) was retained after 90 min of preincubation at temperatures ranging from 5 to 30 °C (Fig. 7D). However, a significant remarkable decrease in EstRag activity (36.78 and 23%) at *P* < 0.05 was noticed after 90 min of preincubation at 35 and 40 °C, respectively.

The profile of EstRag activity in the presence of some metal ions and inhibitors was displayed in Table 2. EstRag activity was significantly enhanced (160.46 ± 0.023 and 391.46 ± 0.009%) at *P* < 0.05 after 30 min of preincubation with 5 mM Ca^2+^and Cu^2+^, respectively (Table 2). After 30 min of preincubation with 5 mM Zn^2+^, K^2+^, and Mo^2+^ separately, EstRag activity decreased significantly (42.883 ± 0.006, 68.71 ± 0.014, and 79.04 ± 0.03%) at *P* < 0.05. Full EstRag activity (100%) was retained after 30 min of preincubation with 5 and 10 mM of Mg^2+^. Preincubation of EstRag with EDTA at 5 and 10 mM for 30 min resulted in significantly enhanced activity of 196 ± 0.026 and 206.74 ± 0.033%, respectively (Table 2). Similarly, after 30 min of preincubation at 5 and 10 mM of β-mercaptoethanol, a significant stimulatory effect on EstRag activity (252.55 ± 0.006 and 225.11 ± 0.053%) was observed (Table 2).

**Table 2:** Effect of some metal ions and inhibitors on EstRag activity

Effector	Residual activity (%) at	
	5 mM	10 mM
Control*	100.00	
Ca^2+^	160.46 ± 0.023	101.50 ± 0.011
Cu^2+^	391.46 ± 0.009	ND
Zn^2+^	42.83 ± 0.006	ND
K^+^	68.71 ± 0.014	7.88 ± 0.005
Mo^2+^	79.04 ± 0.030	49.88 ± 0.015
Mg^2+^	93.44 ± 0.004	121.13 ± 0.059
Mn^2+^	ND	ND
EDTA	196.67 ± 0.026	206.42 ± 0.033
*Β*-mercaptoethanol	252.55 ± 0.006	225.11 ± 0.053

*ND* not detectable

*Without effector

EstRag activity-profile in the presence of some organic solvents and detergents was shown in Table 3. Preincubation of EstRag with 20%(v/v) glycerol and 10%(v/v) diethyl ether for 30 min separately did not exhibit either significant stimulatory or inhibitory effect on enzyme activity. A significant and remarkable decline in EstRag activity (38.91 ± 0.004, 4.29 ± 0.014, 19.25 ± 0.007, and 19.28 ± 0.029%) at *P* < 0.05 was recorded after preincubation for 30 min with 10% (v/v) of ethanol, acetone, isopropanol, and N-butanol, respectively. A significant stimulatory effect at *P* < 0.05 on EstRag activity (121.71 ± 0.032%) was stated after 30 min preincubation with 20%(v/v) DMSO. For hexane and methanol at 20%(v/v) each, a slight significant decrease in EstRag activity (94.21 ± 0.049 and 89.22 ± 0.011%) at *P* < 0.05 was observed, respectively.

**Table 3:** Effect of some organic solvents and detergents on EstRag activity

Organic solvent	Residual activity (%) at		Log P^a^
	10% (v/v)	20% (v/v)	
Control (without treatment)	100.00		
Glycerol	80.93 ± 0.040	97.73 ± 0.009	− 3.180
Diethyl ether	104.53 ± 0.013	ND	0.870
Ethanol	38.91 ± 0.004	15.34 ± 0.012	− 0.310
Hexane	74.04 ± 0.054	94.21 ± 0.049	3.900
Acetone	4.29 ± 0.014	ND	− 0.240
DMSO	83.60 ± 0.004	121.71 ± 0.032	− 1.378
Isopropanol	19.65 ± 0.007	19.65 ± 0,007	0.074
N-butanol	19.28 ± 0.029	ND	0.610
Methanol	89.22 ± 0.011	89.22 ± 0.011	− 0.760

Organic solvent	Residual activity (%) at		Log P^a^
	0.1%(v/v)	0.25%(v/v)	
SDS	118.62 ± 0.00	101.50 ± 0.00	
Triton X-100	93.36 ± 0.015	94.38 ± 0.022	
Tween-20	ND	ND	
Tween-80	89.99 ± 0.027	55.94 ± 0.031	

*ND* not detectable

^a^A quantitative estimate for the polarity of a given solvent. It is the logarithm of a solvent's partitioning coefficient between water and octanol

Estrag activity was fully maintained (100%) after 30 min of preincubation with 0.1 and 0.25% (v/v) SDS (Table 3). However, around 94% of EstRag activity was retained after 30 min of preincubation with 0.1 and 0.25% (v/v) Triton X-100. Conversely, a significant and remarkable decrease in EstRag activity (55.94 ± 0.034%) at *P* < 0.05 was observed after 30 min of preincubation with 0.25%(v/v) Tween-80.

**Fig. 8:**
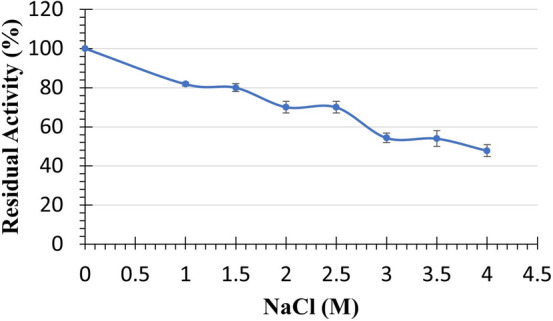
Stability profile of purified to homogeneity EstRag after 30 min preincubation in different concentrations of NaCl. Results are the mean of experimental readings performed in triplicate ± SE (standard error) bars

The effect of NaCl on EstRag activity was demonstrated in Fig. 8. EstRag activity (80 ± 0.019, 70 ± 0.037, and 70 ± 0.02%) was retained after 30 min preincubation with 1.5, 2, and 2.5 M NaCl, respectively. However, higher concentrations of NaCl above 2.5 M to 4 M resulted in a significant decline in EstRag activity (~ 50 ± 0.033).

The kinetic parameters for hydrolysis of the ultimate easily hydrolyzed substrate (*p-*NP-C2) by EstRag were presented in Table 4 and Fig S5. EstRag exhibited substrate affinity (*Km*), catalytic turnover (*Kcat*) and catalytic efficiency (*Kcat/Km*) of 0.031 mM, 20.39 s^−1^ and 657.7 s^−1^. mM^−1^ (Table 4), respectively on *p-*NP-C2, deduced from Lineweaver–Burk plot (Fig S5).

**Table 4:** Substrate specificity of EstRag on *p*-nitrophenyl esters

*p*-NP esters	Specific activity (U/mg)	Relative activity (%)	*Km* (mM)	*Kcat (*sec^−1^)	*Kcat*/*Km* (sec^−1^ mM^−1^)
*p*-NP-C2	0.470 ± 0.0002	100.00	0.031	20.39	657.7
*p*-NP-C4	0.110 ± 0.0008	25.77 ± 0.02	ND	ND	ND
*p*-NP-C6	0.089 ± 0.0003	18.99 ± 0.05	ND	ND	ND
*p*-NP-C8	0.000	0.00	ND	ND	ND
*p*-NP-C12	0.000	0.00	ND	ND	ND

*ND* not detectable

Values are the average of three readings ± standard error (SE)

As displayed in Table 4, the favorable substrate specificity of EstRag towards *p*-NP esters of varied lengths (C2-C12) was investigated. The maximum enzyme activity was perceived in the presence of *p*-NP-C2. The maintained enzyme activity on *p*-NP-C4 and *p*-NP-C6 was 25.77 and 18.99%, respectively, when compared to enzyme activity on *p*-NP-C2 (set as 100%). No enzyme activity could be detected upon using *p*-NP-C8 and *p*-NP-C12 as substrates.

## Discussion

Lipases/esterases are the most significant biocatalysts for industrial applications. There is a growing demand for such enzymes with unique features (e.g., cold-adeptness, detergent stability, organic solvents stability, metal ions stability, etc.) to fit into the rigorous industrial processes with harsh conditions (Romano et al. 2015). In this study, the entire ORF (654 bp) encoding cold- adapted esterase EstRag (217 amino acids) (locus WP_103118438.1 from *Lysinibacillus* sp. YS11 genome sequence NZ_CP026007.1) was selected at random, cloned, heterologously expressed in *E. coli*, and biochemically described. EstRag has a typical α/ß hydrolase fold as depicted in the 3D structural model. EstRag's amino acid sequence phylogeny (Fig. 1) showed that it belongs to the esterase/lipase family VI. Currently, all known esterases/lipases members are categorized into 19 families (I-XIX) based on their amino acid sequences and enzyme characteristics, according to the Arpigany and Jaeger taxonomy (Arpigny and Jaeger 1999). The classification of esterases/lipases by Arpigany and Jaeger was updated by Jaeger and Eggert 2002, Hausmann and Jaeger (2010), Kovacic et al. (2019). According to the ESTHER database's corresponding classification of etserases/lipases, family VI is designated as lysophospholipase carboxylesterase (ESTHER family). Phospholipases and carboxylesterases in this class have broad substrate specificity. Members of family VI exhibit up to 40% sequence similarity with eukaryotic lysophospholipases. BLASTP sequence similarity against the protein data bank (PDB) found that EstRag and 6BJE A (Lysophospholipases of Homo sapiens) share 29.66 percent identity. Based on multiple alignments with esterases received from the PDB database, LOMETS MODELLER chose the PDB entry 1fj2_A (human acyl protein thioesterase) as the best template with the most sequence similarity. The physiological activities of bacterial family VI esterases are still unknown. It is recognized, however, for human acyl-protein thioesterase I, which shares significant sequence and structural similarities with esterases from the family VI. The in vivo functionality of acyl protein thioesterases is S-palmitoylation of cysteine residues in G protein alpha subunits I (Pesaresi and Lamba 2005; Caswell et al. 2022). EstRag's structural and sequence similarities to the PDB template entry 1AUO of *P. fluorescens*, a member of family VI, were determined using TM-align and SAS online programs. EstRag’s multiple sequence alignment with other members of esterase family VI suggested the catalytic triad of EstRag at Ser^110^-Asp^163^-His^194^. Similarly, the crystal structure of esterase of *P. fluorescens* (PDB entry 1AUO) (Kim et al. 1997) and the crystal structure of esterase of human acyl thioesterase I (PDB entry 1fj2_A) (Devedjiev et al. 2000) confirmed the catalytic triad localized at Ser^114^-Asp^168^-His^199^ and Ser^114^-Asp^168^-His^199^, respectively. According to the ESTHER database, there are 27 esterase members of family VI (LYsophospholipase_Carboxylesterase) whose 3D structure has been established experimentally and deposited in the PDB. PDB entries for family VI esterases belong to *P*. *aeruginosa*, *P*. *fluorescens*, *Agrobacterium tumefacien* stran C58, *Dyadobacter fermentans* DSM 18053, *Bacillus cereus*, *Homosapiens*, *Arabidopsis thialana*, and *Zea mays.* However*,* the current number of protein sequences of LYsophospholipase_Carboxylesterase in protein databases is 335 hits form variable sources like bacteria, yeasts, plants, and Homo sapiens, according to the updates of ESTHER database.

Esterases classified in family VI are small proteins ranging from 23 to 26 kDa.The experimental and theoretical EstRag’s molecular weight (around 25 kDa) were in good agreement and harmony with corresponding members of family VI esterases.

EstRag's maximum activity was found at 35 °C, with roughly 37 and 15% of its activity persisting at 20 and 5 °C, respectively (Fig. 7C), indicating its cold-adaptive property. (Santiago et al. 2016). Although EstRag was thermally unstable at temperatures higher than 35 °C, it lost most of its activity after 90 min of incubation (Fig. 7D). These characteristics make EstRag a perfect biocatalyst for application in low temperature processes, contributing not only to energy savings but also to the protection of heat-labile medicinal compounds (Joseph et al. 2007).

The thermal profile of EstRag was in accordance with the previously reported cold-adapted esterases, for instance, EstC (optimal activity at 35 °C and retention of 25% relative activity at 10 °C) (Brault et al. 2012), estHIJ from *Bacillus halodurans* (maximal activity at 30 °C and 15% relative activity at 4 °C, thermal unfolding at 50 °C) (Noby et al. 2019). EstK from *Pseudomonas mondelii* (maximal activity at 40 °C and thermal inactivation at 60 °C) (Hong et al. 2012), rEst97 (highest activity at 35 °C, 25% retained activity after 15 min at 35 °C) (Fu et al. 2013). Cold-lipolytic enzymes, on the other hand, are a valuable resource for unravelling the cold adaptation process of psychrophilic proteins (Siddiqui and Cavicchioli 2006; Kube et al. 2013). Cold-active enzymes, on the whole, have a high catalytic efficiency, although they have limited thermal stability. In most situations, the adaptation to cold is accomplished by a decrease in activation energy, which may result from greater flexibility of either a specific area or the entire protein structure. This increased suppleness appears to be caused by the psychrophilic enzymes' low temperature stability (D’Amico et al. 2002).

Thanks to recent developments in the clarification of the molecular properties of cold-adapted enzymes gained from X-ray crystallography, protein engineering, and biophysical approaches, the adaptation strategies have been recognized. Psychrophilic organisms and their enzymes have piqued the scientific community's interest due to their unique properties, which make them particularly useful in investigating the probable relationship between stability, flexibility, and specific activity, as well as worthy biotechnological tools (D’Amico et al. 2002).

EstRag has lower Gly and Met percentages (6.9 and 2.3%, respectively) than previously reported cold-adapted esterases such as estHIJ (7.6% Gly and 2.4% Met) [15] and rEst97 (13.1% Gly and 3.9% Met) [42]. The impact of these residues could be explained by native flexibility, which is linked to overall protein flexibility and high specific activity at low temperatures (Mavromatis et al. 2002; Fu et al. 2013). EstRag's lower Gly and Met content may explain its superior thermal stability (37% retained activity after 90 min at 35 °C) compared to rEst97 (25% retained activity after 15 min at 35 °C) (Fu et al. 2013). EstRag demonstrated substantially poorer thermostability than estHIJ (70% maintained activity after 6 h at 40 °C) (Noby et al. 2019), although having lower Gly and Met levels than estHIJ. This could be due to the presence of other variables, rather than Gly and Met content, influencing protein flexibility. When compared to their counterparts from mesophilic and thermophilic bacteria, psychrophilic enzymes have a lower Arg/Arg + Lys ratio and a higher Gly and Met content. More ionic interactions (such as salt bridges and hydrogen bonds) are produced by Arg, which contributes to structural rigidity. EstRag had a ratio of 0.357 in this setting, which was low relative to other cold-adaptive esterases previously identified. EstRag's poor thermostability (37% retained activity after 90 min at 35 °C) compared to EstSL3's (40% retained activity after 30 min at 55 °C) could be attributable to EstSL3's high Arg/Arg + Lys ratio (0.62) (Wang et al. 2016). On the other hand, a high Lys residue concentration contributes to protein structural flexibility (Khan and Sylte 2009).

The activity of cold-adapted enzymes at low temperatures could be attributed to the continuous mobility of the enzyme catalytic domains, which reduces activation energy (Noby et al. 2019). However, such flexibility makes the active site heat sensitive and therefore unable to conduct catalysis beyond a certain temperature that causes thermal inactivation (Marx et al. 2007; Barroca et al. 2017). In contrast to cold-adapted enzymes, the stiffness of the active site in their mesophilic and thermophilic counterparts allows them to maintain activity at higher temperatures (Marx et al. 2007). The mesophilic esterases PMGL2 (Petrovskaya et al. 2016) and Est06 (Dukunde et al. 2017), for example, have an optimal activity in the mesophilic range (45–50 °C). Variations in thermal stability could be ascribed to the ratio of flexible residues and their arrangement, whether localized around the active site or dispersed throughout the entire structure (Marx et al. 2007).

EstRag demonstrated considerable activity throughout a wide pH range from 7 to 10, with optimum activity at pH 8.0. (Fig. 7A). The present findings are in accordance with those of Est700 and Est11, which showed optimal activity at pH 8 and 7.5, respectively (Wu et al. 2013; Zhang et al. 2018) (Table 5). In comparison, the ideal Ph of other cold-adapted esterases ranges from 9.0 to 10.5 (LESUISSE et al. 1993; Kanjanavas et al. 2010; Cai et al. 2014; Gricajeva et al. 2016), which is higher than EstRag. Moreover, EstRag was stable over a Ph range from 7 to 10 with around 100% retained relative activity after 20 h of incubation (Fig. 7B), outperforming most reported esterases from other families, such as *Alkalibacterium* sp. EstSL3 (Wang et al. 2016) and *Zunongwangia profunda* EstLiu (Ganasen et al. 2016). The neutral to slight alkaline Ph optima for EstRag could be elucidated by the fact that the enzyme is most probably secreted in the cytoplasm internally, which has a lower Ph than the external environment (Krulwich et al. 1997). This localization was further confirmed by the absence of a signal peptide in EstRag and estHIJ [19]. EstRag’s appropriateness for working efficiently in extreme alkaline settings in industrial processes is influenced by alkaline Ph optima and stability.

**Table 5:** Comparison between different reported cold-adapted esterases concerning biochemical properties

Microbial source	Enzyme designation	Expression host	Amino acid length	Optimal pH	Optimal temperature (^o^C)	Metal ion resistance	Detergent resistance	Molecular weight (kDa)	Family	Reference
*Lysinibacillus* sp.	EstRag	*E. coli* BL21 (DE3) Rosetta	217	8.0	35	Significantly augmented 3 times fold its normal activity in the presence of copper ions	Strongly enhanced by SDS Resistant to triton X-100	25	New member of family VI	This study
*Psychrobacter pacificensis*	Est11	*E. coli* BL21 (DE3)	297	7.5	25	Inhibited by Cu^2+^ and Zn^2+^ Resistant to Mg^2+^, Ca^2+^ and Mn^2+^	Resistant to tween 20, tween 80 and triton X-100	32.9	New family	Wu et al. (2015)
*Pseudomonas mandelii*	EstK	*E. coli*		8.5	40	Inhibited by Cu^2+^ Resistant to Ca^2+^, K^+^ and Mg^2+^	Not tested	33	Not mentioned	Hong et al. (2012)
*Bacillus halodurans*	estHIJ	*E. coli* BL21 (DE3) Rosetta	248	7–8	30	Na^+^, K^+^, Mg^2+^, and Ca^2+^ resistant	Inhibited by SDS Resistant to tween 20, tween 80 and triton X-100	29	XIII	Noby et al. (2019)
*Bacillus licheniformis*	Est700	*E. coli* BL21 (DE3)	208	8.0	30	Resistant to Mg^2+^, Ca.^2+^ Ba^2+^, Mn^2+^, Na^+^ and K^+^	Inhibited by SDS Resistant to tween 20, tween 80 and triton X-100	25	I	Zhang et al. (2018)
*Bacillus cohnii* strain N1	EstN7	*E. coli* BL21 (DE3) Rosetta	320	9.0	5	Inhibited by Cu^2+^ and Zn^2+^ Resistant to Mn^2+^, K^+^, Na^+^ and Ca^2+^	Inhibited by SDS Resistant to tween 20, tween 80 and triton X-100	37	IV	Noby et al. (2018)
*Salinisphaera* sp. P7-4	P7-4 esterase	*E. coli* BL21 (DE3)	316	8.0–9.0	25	Inhibited by Cu,^2+^ Ni^2+^ and Zn^2+^ Resistant to Ca^2+^, K^+^ and Mg^2+^	Not tested	34.4	Not mentioned	Kim et al. (2011)
*Psychrobacter cryohalolentis* K5T	EstPc	*E. coli* BL21 (DE3)	315	8.5	35	Inhibited by Cu^2+^ and Zn^2+^ Resistant to Mg^2+^, Mn^2+^ and Co^2+^	Inhibited by SDS Resistant to tween 20,and triton X-100	33	V	Novototskaya-Vlasova et al. (2012)
*Pseudoalteromonas* sp. strain 643A	EstA	*E. coli* TOP10F’	207	8.0	35	Inhibited Zn^2+^, Mg^2+^, Co^2+^ and Cu^2+^ Strongly activated by Ca^2+^	Not tested	23	GDSL family of lipolytic enzymes	Cieśliński et al. (2007)
*Pseudoalteromonas arctica*	EstO	*E. coli* TunerTM (DE3)	400	7.5	25	Completely inhibited by Al^2+^, Cu^2+^, Fe^2+^, Cr^2+^, and Co2, whereas Ca^2+^, Mg^2+^, Se^2+^ and Mn^2+^ had no or only minor effect	Completely inhibited by SDS and Tween 20	44.1	Serine hydrolase family	Khudary et al. (2010)
*Deinococcus radiodurans*	EstDR4	*E. coli*	312	8.0	30	Resistant to Li^+^, Na^+^, K^+^, and Mg^2+^ significantly inhibited by Co^2+^, Cu^2+^ and Zn^2+^	Significantly activated by Tween 80 and Triton X-100 inhibited by SDS and Tween 20	33	IV	Zhang et al. (2021)
*Streptomyces coelicolor* A3(2)	EstC	*E. coli* BL21 (DE3)	327	8.5–9.0	35	Significantly inhibited by Cu^2+^, Zn^2+^, Ni^2+^, Fe^2+^ and Mn^2+^ Resistant to Ca ^2+^ and Mg^2+^	Not tested	35	v	Brault et al. (2012)
*Acinetobacter venetians* V28	V28 esterase	*E. coli* BL21 (DE3)	338	9.0	40	No significant inhibition of activity was obtained with Ca^2+^, Cu^2+^, Co^2+^, Cd^2+^, Mg^2+^, K^+^, Mn^2+^ and Zn^2+^	Resistant to Tween 20. Tween 80 and SDS	35	Not mentioned	Kim et al. (2012)
*Microbulbifer thermotolerans*	MtEst45	*E. coli* BL21 (DE3)	495	8.17	46.2	Strongly inhibited by Hg^2+^, Zn^2+^, and Cu^2+^	Not tested	45.5	III	Lee (2016)
*Microbulbifer thermotolerans* DAU221	CEST	*E. coli*	307	8.0	15	Enzyme activity was increased by Na^+^and Mg^2+^ ions but was strongly inhibited by Cu^2+^ and Hg^2+^ ions	Not tested	31.24	VI	Lee et al. (2014)
Metagenomic library	Est97	*E. coli*	247	7.5	35	Strongly inhibited by Zn^2+^, and Cu^2+^ Resistant to Ca^2+^ and Mg^2+^	Inhibited by SDS and Tween 20	26.9	VIII	Fu et al. (2013)

The metal ions Zn^2+^ and Mn^2+^ were shown to severely inhibit EstRag activity. Zn^2+^ has been reported to strongly inhibit a variety of esterases, including estHIJ (Noby et al. 2019), EstF (Fu et al. 2011), Est97(Fu et al. 2013), EstA (Cieśliński et al. 2007) and EstC (Brault et al. 2012), but the common mechanism of Zn^2+^ inhibitory impact on esterases remains unknown and needs to be investigated in the future. Wherase EstRag activity significantly increased in the presence of Mg^2+^ and Ca^2+^ ions. The Ca^2+^ is generally thought to be required for lipase and esterase activation by strengthening protein structure and decreasing product inhibition (Guncheva and Zhiryakova 2011). Incredibly EstRag activity was greatly augmented four times fold its normal activity in the presence of copper which is a unique characteristic for EstRag. On the contrary, other cold- adapted esterases reported in the literature such as: EstDR4 (Zhang et al. 2021), EstN7 (Noby et al. 2018), Est11(Wu et al. 2015), P7-4 esterase (Kim et al. 2011), 643A esterase (Cieśliński et al. 2007) and EstPc (Novototskaya-Vlasova et al. 2012) were all strongly inhibited by the action of copper (Table 5). However, in the presence of Cu^2+^, some counterparts of esterases from mesophilic and thermophilic microorganisms showed either very slight inhibition (around 94% retained activity) or full resistance (100% retained activity), such as EstATII (Mohamed et al. 2013), LKE-028 (Kumar et al. 2012), EstR (Quyen et al. 2007), EstA (Chu et al. 2008) and EstEH112 (Oh et al. 2012). EstRag's significantly enhanced activity in the presence of Cu^2+^ is a unique property that has yet to be seen in other esterases. In a prospective investigation, the formation of the EstRag-Cu^2+^ complex should be explored in terms of the position and type of amino acids that contribute to this complex’s formation. In a future investigation, crystallizing EstRag in the presence of Cu^2+^ is strongly suggested. The amazing activity of significantly enhanced EstRag in presence of Cu^2+^ is an extraordinary property that would underpin its potential for bioremediation of oil contaminated water and soil with a high Cu^2+^ load.

Incredibly, the metal chelator EDTA showed a discernible stimulatory effect on EstRag activity, demonstrating that EstRag is not a metalloenzyme. Similarly, EstCS1 (Park et al. 2020), and Est2L/Est4L (Park et al. 2021) were reported as non-metalloesterases (no detectible inhibitory effect by EDTA at 10 mM). Likewise, β-mercaptoethanol significantly enhanced EstRag activity by 2.5 times fold at concentrations of 5 and 10 mM. On the other hand, β-mercaptoethanol showed neither inhibitory nor stimulatory effects on EstN7 (Noby et al. 2018), estHIJ (Noby et al. 2019) and esterase of *Salimicrobium* sp. LY19 (Xin and Hui-Ying 2013) enzyme activity (Table 5).

In the case of detergents, EstRag was obviously enhanced by 1.18 times its initial activity in the presence of SDS at a concentration of 0.25%. This might be attributed to the fact that SDS acts as an activator by enhancing substrate solubility, stabilizing enzyme conformation, improving the availability of substrates to the active core linked with the hydrophobic binding, and limiting protein aggregation (Guncheva and Zhiryakova 2011). Conversely, SDS had a strong inhibitory effect on EstN7, estHIJ, Est11, Est700 and EstPC (Novototskaya-Vlasova et al. 2012; Wu et al. 2015; Noby et al. 2018, 2019; Zhang et al. 2018) (Table 5). Additionally, EstRag retained more than 90% of its activity in the presence of Triton X-100 at both concentrations, which is in accordance with previously reported findings of EstN7 and estHIJ (Noby et al. 2018, 2019). EstRag's detergent stability is a promising attribute that would suggest its likely usage in the detergent industry.

EstRag remained either moderately stable or dramatically deactivated in non-polar hydrophobic liquids. Due to its moderate stability in non-polar hydrophobic organic solvents such as hexane and diethyl ether for 30 min at 10%(v/v), EstRag would be used in esterification and trans-esterification processes that are often carried out in low-water-content media utilizing non-polar solvents (Guncheva and Zhiryakova 2011). So far, several esterases with tolerance to organic solvents have been outlined, including Est11 (tolerant to glycerol, ethanol, DMSO, and isopropanol) (Wu et al. 2015), Est700 (tolerant to n-hexane, n-heptane, xylene, isopropanol, and ethanol) (Zhang et al. 2018) Estpc and (tolerant to DMSO and methanol) (Novototskaya-Vlasova et al. 2012). Significant EstRag inactivation in the presence of polar organic solvents, such as ethanol, acetone, and isopropanol may be attributed to the removal of critical bound-water monolayer from the enzyme molecule, which is required for its activity (Ogino and Ishikawa 2001). Though the sensitivity of esterases to solvents differs, polar solvents tend to induce more severe enzyme inactivation than non-polar solvents (Doukyu and Ogino 2010). However, the appreciable stability of EstRag in non-polar organic solvents like hexane and diethyl ether may be due to the interaction between the non-polar organic solvents and the hydrophobic amino acids existing in the lid that shield the enzyme’s catalytic site and keep it open, allowing it to catalyze (Rúa et al. 1993). The better stability of EstRag in hexane compared to that in diethyl ethyl may be attributed to the high log P value of hexane. Similar findings were observed in some halophilic lipases and the esterase from *Salimicrobium* sp. LY19 (Dheeman et al. 2011; Xin and Hui-Ying 2013).

The activity of EstRag following incubation with NaCl was evaluated to see if it possessed another habitat-specific trait, namely salt-tolerance. In the present findings, EstRag displayed good stability to some extent in the presence of NaCl as it maintained more than 70% of its initial activity at concentrations of up to 2 M of NaCl. This good halotolerance makes EstRag a useful biocatalyst for high-salt processes like cheese ripening and enhancing the flavor of pickled food (Esteban-Torres et al. 2014). EstRag salt tolerance is comparable to that of the esterase EstKT4 (1.0 M) (Esteban-Torres et al. 2014), but it is less than that of Est10 and Est11 (5.0 M) (Wu et al. 2013, 2015).

EstRag’s preferential substrate specificity towards short chain p-nitrophenyl substrates (p-NP-C2 >  > p-NP-C4 >  > p-NP-C6) is a feature of esterase of family VI (Bornscheuer 2002). This would entail the urgent need to expand EstRag's primary substrate binding region using directed evolution approaches in future research.

The different acyl chain lengths of p-NP-esters were used to measure the kinetic characteristics of EstRag (C2, C4, C6, C8 and C12). Generally speaking, low *Km* and high *Kcat* values do indicate that an enzyme has a high affinity for the substrate being employed. Also, The higher the *Kcat*/*Km* values the more specific the enzyme is for that substrate. Our results showed that EstRag had a strong affinity towards pNP-C2 (*Km* = 0.031 mM) with also relatively high catalytic efficiency (*Kcat/Km* = 657.7 mM^−1^ pNP-C2 compared to other reported esterases (Table 6). The Km value was much lower than that of estHIJ, Est700, EstC, EstSL3 and CEST (Noby et al. 2019; Zhang et al. 2018; Brault et al. 2012; Wang et al. 2016; Lee et al. 2014) which demonstrated that EstRag had better affinity and preference for the substrate (p-NP-C2). However, not detectable *kcat*/*Km* values of EstRag toward longer chain pNP esters (C4, C6, C8 and C12) suggest that they might not be natural substrates of EstRag. Although esterases have different substrate specificity for pNP ester substrates, our results are in accordance with that of other reported cold adapted esterases shown in Table 6. These findings imply that EstRag may prove to be an extremely valuable biocatalyst for the commercial production of volatile short chain esters, such as flavors.

**Table 6:** Values of *Km* and *Kcat* of some previously reported esterases

Enzyme	*Km* (mM)	*Kcat* (s^−1^)	*Kcat/Km* (s^−1^ mM^−1)^	Substrate	Reference
EstRag	0.031	20.39	657.7	*p*-NP-C2	This study
EstHIJ	0.10	78.00	780.41	*p*-NP-C2	Noby et al. (2019)
Est11	0.034	5.75	169.11	*p*-NP-C4	Wu et al. (2015)
Est700	2.11	78.80	37.39	*p*-NP-C2	Zhang et al. (2018)
EstDR4	0.3725	28.34	76.08	*p*-NP-C8	Zhang et al. (2021)
EstC	2.90	451.00	156.00	*p-*NP-C2	Brault et al. (2012)
EstSL3	0.15	307.69	2051.26	*p*-NP -C2	Wang et al. (2016)
CEST	0.278	1.90	6.83	*p*-NP-C2	Lee et al. (2014)

## Conclusion

In this study, a novel member of the rare family VI esterases (LYsophospholipase_Carboxylesterase) was cloned from *Lysinibacillus* sp. YS11, heterologously overexpressed in *E. coli*, and biochemically characterized for the first time ever. The novel esterase, designated EstRag is cold-adaptive, detergent stable, and Cu^2+^ resistant. Promising features of EstRag would suggest its potential for exploitation in industrial processes conducted under harsh conditions involving low temperatures, high loads of detergents and Cu^2+^. EstRag is regarded as a value-added venue for esterases, a large and important category of industrial enzymes.

## Supplementary Information

Below is the link to the electronic supplementary material.

Supplementary file1 (TIF 300 kb)—Ramachandran plot generated by PROCHECK for the 3D predicted model of EstRag. A: the initial predicted 3D model of EstRag. B: the refined 3D model of EstRag

Supplementary file2 (TIF 341 kb)—Verify 3D for the predicted 3D model of EstRag. A: initial predicted 3D model of EstRag. B. refined predicted 3D model of EstRag

Supplementary file3 (TIF 706 kb)—ERRAT graph for the predicted 3D model of EstRag. A: initial predicted 3D model of EstRag. B: refined predicted 3D model of EstRag

Supplementary file4 (TIF 653 kb)—Output of ProSA-web z-score analysis for initial and refined 3D models of EstRag. ProSA-web z-score plot for initial model (A) and refined model (D). Ribbon view of initial model (C) and refined model (F) with lowest energy regions (blue color) and highest energy regions (red color). Energy plot for initial model (B) and refined model €

Supplementary file5 (TIFF 325 kb)—Lineweaver-Burk plot of purified to homogeneity EstRag using p-NP-C2 as a substrate

Supplementary file6 (DOCX 13 kb)
