# Supplementary figures and images for: Molecular study on recombinant cold-adapted, detergent- and alkali stable esterase (EstRag) from Lysinibacillus sp.: a member of family VI
Source: World J Microbiol Biotechnol. 2022 Sep 7;38(12):217. doi: 10.1007/s11274-022-03402-5 (PMC9452428; doi:10.1007/s11274-022-03402-5)

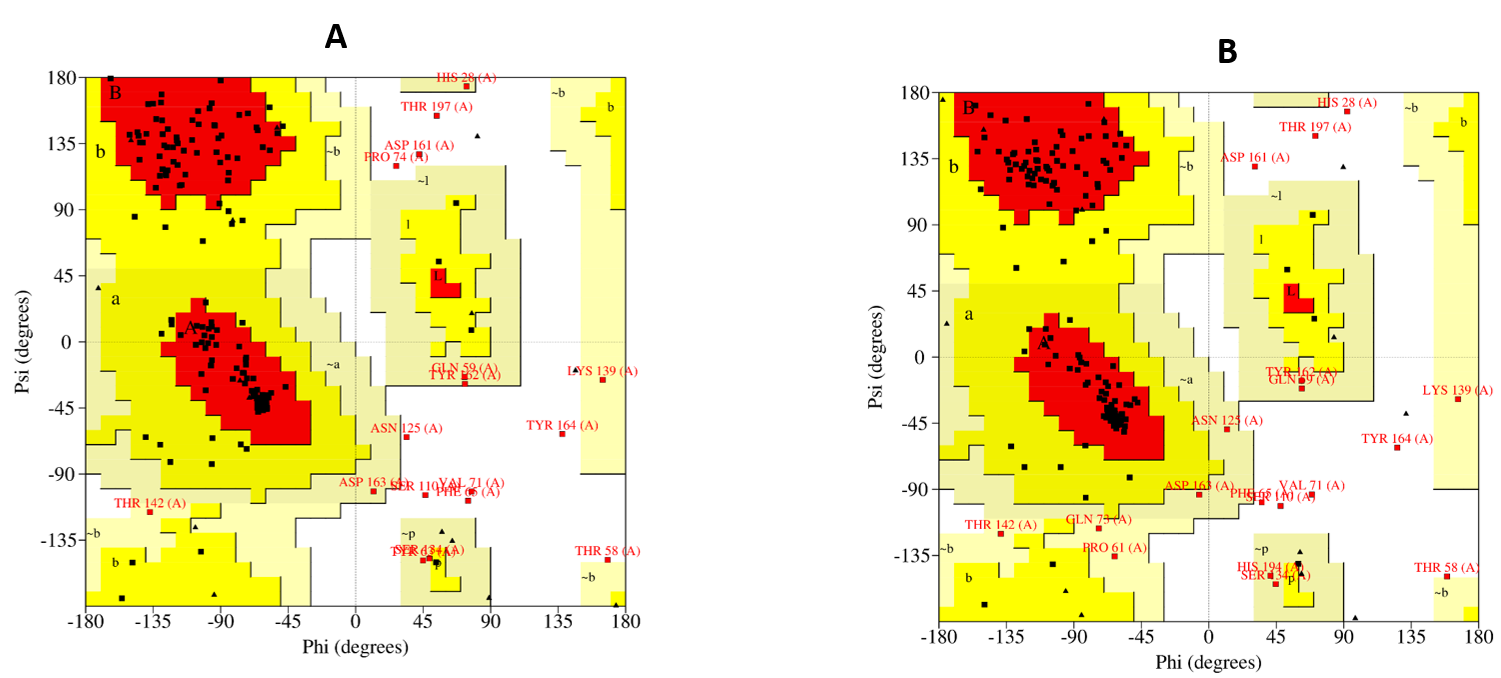

Supplement: Supplementary file 1 — Supplementary file1 (TIF 300 kb)—Ramachandran plot generated by PROCHECK for the 3D predicted model of EstRag. A: the initial predicted 3D model of EstRag. B: the refined 3D model of EstRag [file 11274_2022_3402_MOESM1_ESM.tif]

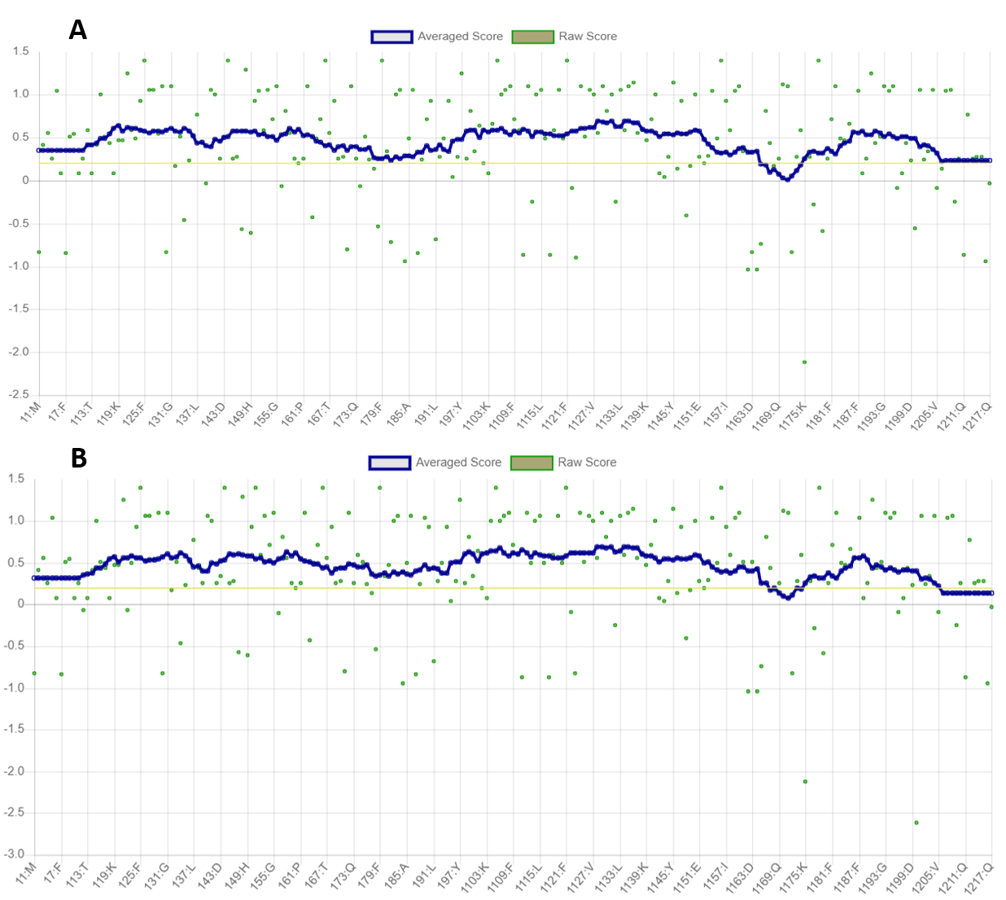

Supplement: Supplementary file 2 — Supplementary file2 (TIF 341 kb)—Verify 3D for the predicted 3D model of EstRag. A: initial predicted 3D model of EstRag. B. refined predicted 3D model of EstRag [file 11274_2022_3402_MOESM2_ESM.tif]

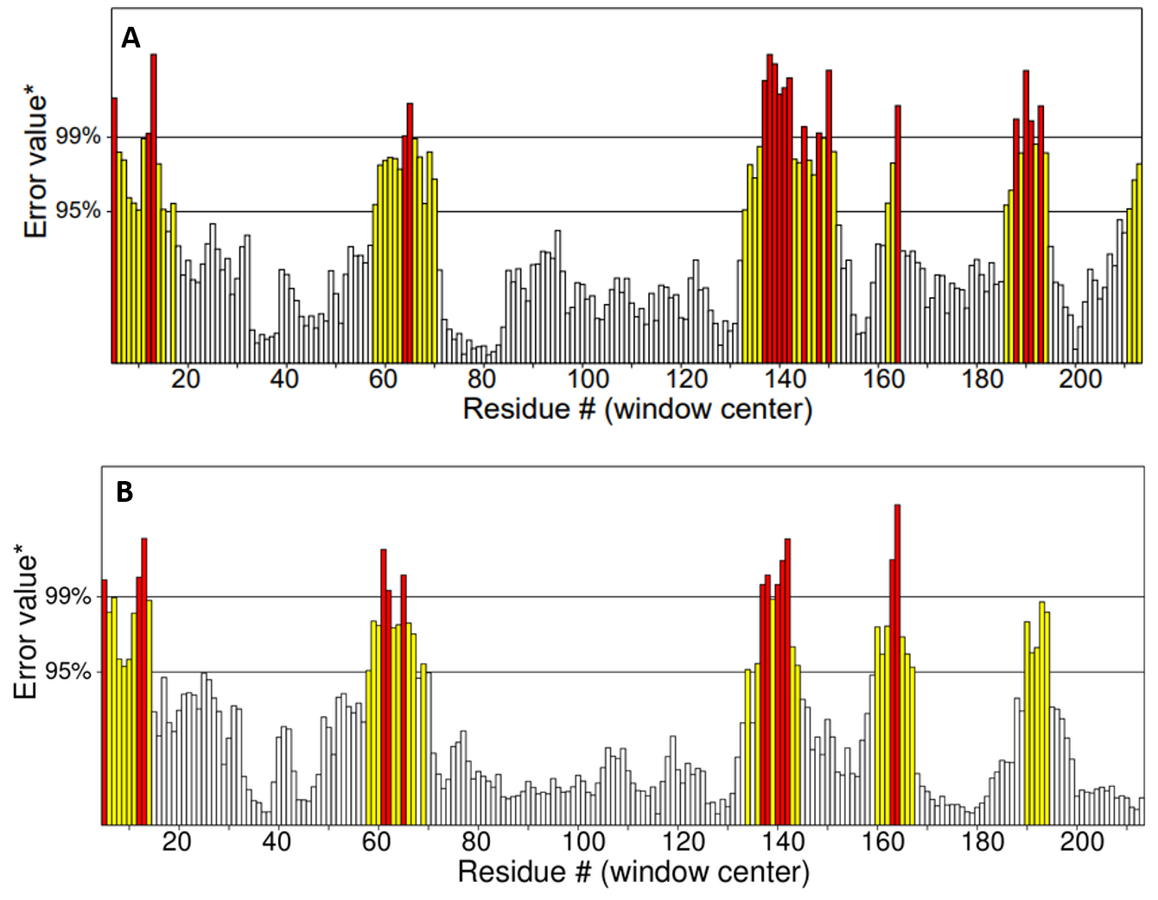

Supplement: Supplementary file 3 — Supplementary file3 (TIF 706 kb)—ERRAT graph for the predicted 3D model of EstRag. A: initial predicted 3D model of EstRag. B: refined predicted 3D model of EstRag [file 11274_2022_3402_MOESM3_ESM.tif]

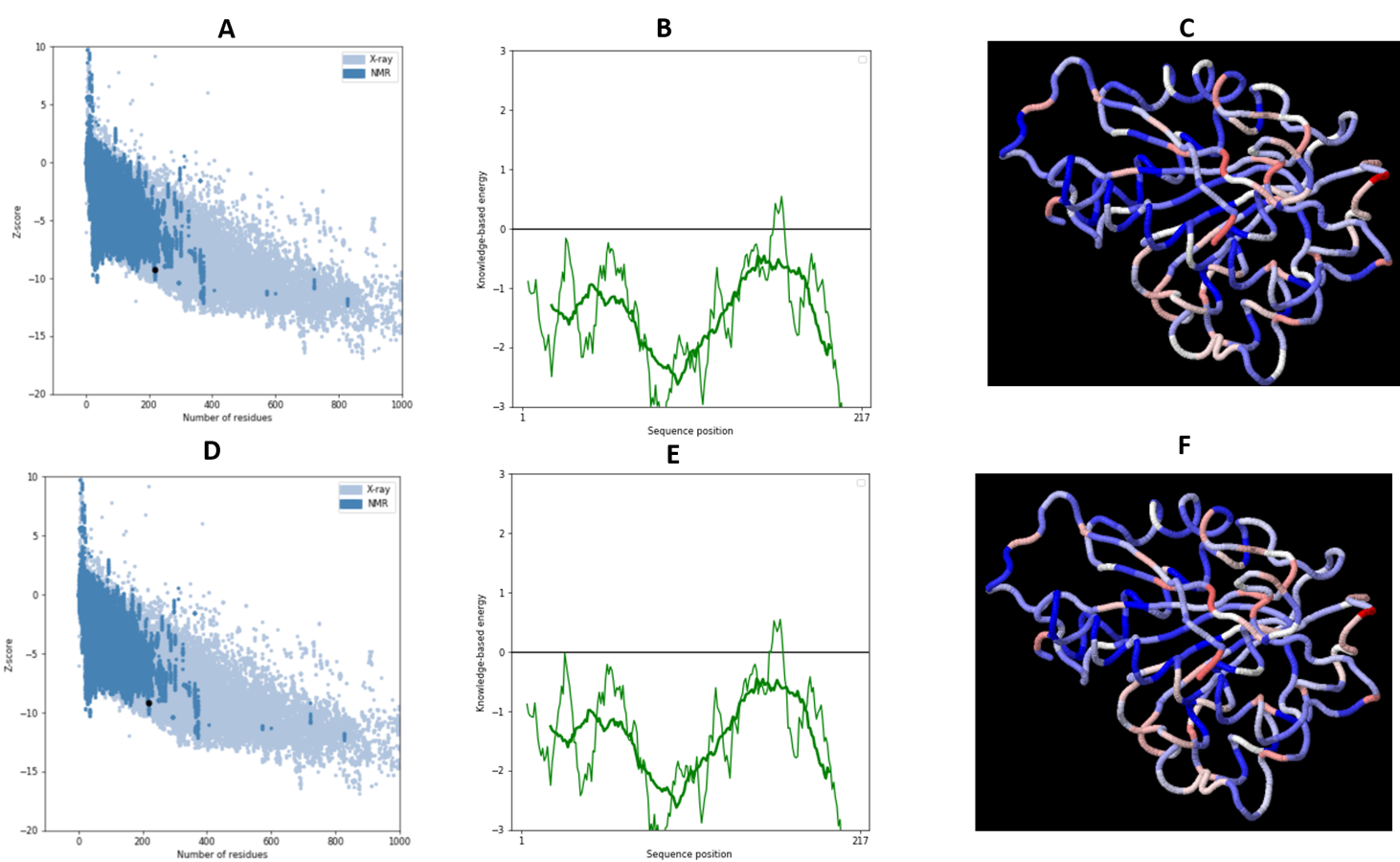

Supplement: Supplementary file 4 — Supplementary file4 (TIF 653 kb)—Output of ProSA-web z-score analysis for initial and refined 3D models of EstRag. ProSA-web z-score plot for initial model (A) and refined model (D). Ribbon view of initial model (C) and refined model (F) with lowest energy regions (blue color) and highest energy regions (red color). Energy plot for initial model (B) and refined model € [file 11274_2022_3402_MOESM4_ESM.tif]
