# Supplementary material for: Molecular study on recombinant cold-adapted, detergent- and alkali stable esterase (EstRag) from Lysinibacillus sp.: a member of family VI
Source: World J Microbiol Biotechnol. 2022 Sep 7;38(12):217. doi: 10.1007/s11274-022-03402-5 (PMC9452428; doi:10.1007/s11274-022-03402-5)
Supplement: Supplementary file 6 — Supplementary file6 (DOCX 13 kb) [file 11274_2022_3402_MOESM6_ESM.docx]

**Supplementary file (S1)**

Analyses of Ramachandran plots through PROCHECK revealed that the initial 3D model had 147 (78.6%) residues in the most favored region, 24 (12.8%) in the additional allowed region, 8 (4.3%) in the generously allowed region, and 8 (4.3%) in the disallowed region (Fig. S1A). However, the refined 3D model had 151 (80.7%) residues in the most favored region, 19 (10.2%) in the addition-*/6al allowed region, 9 (3.8%) in the generously allowed region, and 8 (4.3%) in the disallowed region (Fig. S1B). The analysis of the initial 3D model structure by Verify 3D evidenced that 95.39% of the residues had scores ≥ 0.2 in the 3D/1D score (Fig. S2A), however the refined 3D modeled structure had 91.24% of the residues with scores ≥ 0.2 in the 3D/1D score (Fig. S2B). ERRAT demonstrated that the overall quality factors for the initial and refined 3D structure models were 71.77 and 78.95, respectively (Fig. S3). The analyses of the initial and refined 3D structure models through ProSA-web indicated that the z-scores were 9.22 (Fig.S4A) and 9.12 (Fig.S4D), respectively. The z-score of both models underpinned that they were within the same range of z-scores of other experimentally determined protein chains of similar size in the current PDB. Consequently, this would imply the validity and reliability of the 3D model structure. The energy plot demonstrates the model quality by plotting energies in relation to the sequence position of amino acids. As a rule of thumb, positive values evidence the problematic part of a model. A comparable energy plot for the initial (Fig.S4B, C) and refined (Fig.S4E, F) 3D model structures was presented. The superimposition of the 3D structures of the refined EstRag model and the structure template with PDB entry 1AUO_A, was portrayed in Fig. 5. The root mean square deviation (RMSD) of the superimposed protein structures was 2.14 (204 atoms in refined 3D structure model of EstRag aligned to 204 atoms in 3D structure template model 1AUO_A), likewise indicating a good structure similarity. Additionally, the TM-score was 0.85, localized in the range of 0<TM<1.0 that would indicate that the two superposed proteins were in about the same fold.
